# Safety and Efficacy of Immune Checkpoint Inhibitors for Patients With Metastatic Urothelial Carcinoma and End-Stage Renal Disease: Experiences From Real-World Practice

**DOI:** 10.3389/fonc.2020.584834

**Published:** 2020-11-27

**Authors:** Ming-Chun Kuo, Po-Jung Su, Chun-Chieh Huang, Hao-Lun Luo, Tai-Jan Chiu, Shau-Hsuan Li, Chia-Che Wu, Ting-Ting Liu, Yuan-Tso Cheng, Chih-Hsiung Kang, Yu-Li Su

**Affiliations:** ^1^ Division of Hematology Oncology, Department of Internal Medicine, Kaohsiung Chang Gung Memorial Hospital and Chang Gung University, College of Medicine, Kaohsiung, Taiwan; ^2^ Division of Hematology Oncology, Chang Gung Memorial Hospital at Linkou and College of Medicine, Chang Gung University, Tao-Yuan, Taiwan; ^3^ Department of Radiation Oncology, Kaohsiung Chang Gung Memorial Hospital and Chang Gung University, College of Medicine, Kaohsiung, Taiwan; ^4^ Department of Urology, Kaohsiung Chang Gung Memorial Hospital and Chang Gung University, College of Medicine, Kaohsiung, Taiwan; ^5^ Department of Pathology, Kaohsiung Chang Gung Memorial Hospital and Chang Gung University, College of Medicine, Kaohsiung, Taiwan; ^6^ Clinical Trial Center, Kaohsiung Chang Gung Memorial Hospital, Kaohsiung, Taiwan

**Keywords:** immune checkpoint inhibitor, end-stage renal disease, metastatic urothelial carcinoma, safety, survival

## Abstract

**Background:**

Immune checkpoint inhibitors (ICIs) are used widely for treating metastatic urothelial carcinoma (mUC). In practical settings, evidence is lacking on the efficacy of ICIs in some difficult-to-treat patients, such as those with end-stage renal disease (ESRD). Herein, we evaluate the safety and efficacy of ICIs for patients with mUC and ESRD.

**Methods:**

For this retrospective study, patients with mUC who were given ICIs at Kaohsiung Chang Gang Memorial Hospital and Linkou Chang Gung Memorial Hospital between April 2016 and November 2019 were consecutively enrolled. All clinicopathologic data, treatment responses, and adverse events were recorded. The immune-related adverse events (AEs), objective response rate (ORR), progression-free survival (PFS), and overall survival (OS) were compared between ESRD and non-ESRD groups.

**Results:**

In total, 129 patients with mUC were enrolled, with 11 patients categorized as the ESRD group. Among these patients with ESRD receiving ICIs, 7 of 11 (63.6%) had high-grade (grade ≥3) AEs, chiefly hematologic toxicity. Some rarely encountered AEs were noted, including toxic epidermal necrolysis, tuberculosis reactivation, ascites, and cytokine release syndrome. Patients in the ESRD group had numerically higher ORR (54.5% vs. 28.8%, p = 0.09), PFS (7.1 vs. 3.5 months, p = 0.42), and OS (not reached vs. 15.4 months) than the non-ESRD group. A multivariate Cox regression model demonstrated that leukocytosis (hazard ratio [HR]: 2.63; 95% confidence interval [CI]: 1.23–5.63; p = 0.01) and neutrophil-to-lymphocyte ratio (HR 2.91; 95% CI: 1.30–6.53; p = 0.01) were independent prognostic factors.

**Conclusion:**

Administration of ICIs in patients with mUC and ESRD demonstrated a modest antitumor activity, and should be used with caution for increasing risk of hematologic toxicity.

## Introduction

Urothelial carcinoma (UC) is a common cancer worldwide, with approximately 500,000 new cases diagnosed annually and an estimated 150,000 cancer-related deaths (1). Early-stage UC can be cured through radical surgery, including cystectomy for bladder cancer and nephroureterectomy for upper tract urothelial carcinoma (UTUC). Nevertheless, approximately 10–30% of these patients experience local recurrence or distant metastasis, leading to mortality from such diseases (2). Cisplatin-based chemotherapy has been the gold standard therapy since 1990, with an objective response rate (ORR) of 40–50% and an overall survival (OS) of 14–15 months (3). As the recent breakthrough of immune checkpoint inhibitors (ICIs) has been widely studied for various cancer types, the paradigm of treatment has shifted to ICIs for patients failing to respond to platinum-based chemotherapy and those who are ineligible for cisplatin (4–8). In the pivotal phase 3 KEYNOTE-045 study, compared with conventional chemotherapy, pembrolizumab conferred a significant survival benefit on patients with metastatic UC (mUC) whose conditions were refractory to first-line platinum-based chemotherapy, regardless of the patients’ PD-L1 expression (4). At this time, five ICIs have been approved by the U.S. Food and Drug Administration (FDA) for mUC treatment.

The efficacy of cisplatin-based chemotherapy in patients with mUC is generally limited by poor Eastern Cooperative Oncology Group (ECOG) performance status or chronic kidney disease. In general, the proportion of patients for whom cisplatin is unsuitable may be 30–50% of the population with stage IV mUC (9). Given their more favorable toxicity profile, ICIs have been investigated as first-line treatments for cisplatin-ineligible patients with mUC. The promising OS results from the IMVigor 210 trial demonstrated that atezolizumab monotherapy provided an excellent OS of 15.8 months, prompting the FDA to grant accelerated approval for ICIs as first-line treatment for cisplatin-ineligible patients with mUC (10). However, many patients have been excluded from prospective trials owing to poor ECOG performance status or having coexisting autoimmune disease or end-stage renal disease (ESRD) requiring hemodialysis. Treatment options for patients with such rare conditions remain uncertain, and related evidence is lacking.

ESRD is a common comorbidity in patients with mUC. UTUC and urothelial carcinoma of the bladder (UCB) independently increase the risk of ESRD, with hazard ratios (HRs) for ESRD up to 7.75 and 3.12 in patients with UTUC and UCB, respectively (11). Patients with ESRD, especially women aged 50 to 60 years, also have a high risk of developing UC (12). As ICIs are eliminated through the reticuloendothelial system and are not excreted through renal filtration, their use in patients receiving dialysis provides an alternative therapeutic choice to avoid cumulative toxicity from conventional chemotherapy (13). Only small case series have provided evidence of the safety and efficacy of ICIs in patients with ESRD, and most of such studies have been on melanoma, lung cancer, and renal cell carcinoma (14, 15). To assist such difficult-to-treat patients, data on the safety and efficacy of ICIs are urgently required. The aim of this retrospective study was to evaluate the safety and efficacy of immune ICIs in patients with mUC and ESRD.

## Methods

### Patients

We retrospectively reviewed patients with mUC who received ICIs between April 2016 and November 2019 at Kaohsiung Chang Gung Memorial Hospital and Linkou Chang Gung Memorial Hospital in Taiwan. All clinicopathologic data were collected from electrical medical recording systems by physicians and trained assistants. Database variables included age, sex, ECOG performance status, primary tumor site, visceral or lymph node metastasis, PD-L1 expression by tumor proportion score, ICI type, regimen of combination treatment or previous systemic treatment, laboratory data, treatment response, and adverse events (AEs). The study was approved by the Institutional Review Board of Chang Gung Medical Foundation.

### Treatment

All patients received an anti-PD-1 (nivolumab, pembrolizumab) or anti-PD-L1 (atezolizumab, durvalumab, or avelumab) medication. The regimen, treatment sequence, and combined treatment regimen were at the discretion of the physician. The regimen of combined treatment included chemotherapy, a cytotoxic T-lymphocyte antigen 4 (CTLA-4) inhibitor, and a poly ADP-ribose polymerase (PARP) inhibitor.

### Response Evaluation and Endpoints

All patients had attended scheduled appointments during treatment until disease progression, treatment intolerance, or death. The follow-up visit procedures included physical examinations, laboratory tests, and imaging studies. Patients were subjected to computed tomography scans of the chest or abdomen for tumor response assessments using the Response Evaluation Criteria in Solid Tumors (version 1.1).

The primary endpoint was treatment-related AEs in patients with ESRD. The observed AEs during any round of ICIs were graded according to the Common Terminology Criteria for Adverse Events (CTCAE) version 4.0 (**Supplementary Table 1**). All patients who received at least one cycle of immunotherapy were included in the analysis. The secondary endpoints of the study were treatment response, OS, and progression-free survival (PFS). OS was defined as the time interval from the date of ICIs commencement (any cycle) to the date of death or final patient contact.

### Statistical Analysis

All statistical analyses were performed using SPSS version 21.0 (SPSS Inc., Chicago, IL, USA), and survival curves were plotted using GraphPad Prism version 6.04 (GraphPad Software, La Jolla California, USA). The differences between the ESRD subgroup and patients without ESRD were examined using chi-squared (χ^2^) and t tests for categorical and continuous variables, respectively. We constructed OS and PFS curves using the Kaplan–Meier method. Univariate and multivariate analyses were performed using the Cox proportional hazards regression analysis. A p value <0.05 was considered statistically significant.

## Results

### Patient Characteristics

In total, 129 patients were included in this study, including 11 patients (8.5%) with ESRD who were on maintenance hemodialysis; they were categorized into the ESRD group. Basic patient characteristics are shown in **Table 1**. According to group comparison, the ESRD group had a significantly higher proportion of patients with an ECOG scale score of ≥2 (45.5 vs. 16.1%, p = 0.05), UTUC (72.7% vs. 59.3%, p = 0.05), and anemia (90.0 vs. 35.1%, p = 0.001). No significant difference was noted in age, gender, site of visceral metastasis, tumor proportion score, regimen and sequence of ICIs, white blood cell count, and neutrophil to lymphocyte ratio (NLR) between the two groups. Two-thirds of patients (65.1%) were given anti-PD-1 therapy, and the majority of ICIs were used as monotherapy (64.3%) and as a first-line treatment (75.2%). The individual details of the ESRD group are listed in **Table 2**.

**Table 1:** Patients demographics and baseline characteristics.

	N (%)	ESRD (%)	Non-ESRD (%)	*p* value
N	129	11	118	
Age (median, years)	66	64	66	0.55
Male	76 (58.9)	4 (36.4)	72 (61.0)	0.2
Tumor location				0.05
UCB	49 (38.0)	2 (18.2)	47 (39.8)	
UTUC	78 (60.5)	8 (72.7)	70 (59.3)	
Multifocal	2 (1.5)	1 (9.1)	1 (0.8)	
ECOG				0.05
0-1	102 (79.1)	6 (54.5)	96 (81.4)	
≧2	24 (18.6)	5 (45.5)	19 (16.1)	
Missing	3 (2.3)	0	3 (2.5)	
ICI sequence				0.25
1st line	97 (75.2)	6 (54.5)	91 (77.1)	
2nd line	19 (14.7)	3 (27.3)	16 (13.6)	
3rd line or later	13 (10.1)	2 (18.2)	11 (9.3)	
ICI type				0.75
Anti-PD-1	84 (65.1)	8 (72.7)	76 (64.4)	
Anti-PD-L1	45 (34.9)	3 (27.3)	42 (35.6)	
Treatment partner				0.63
Monotherapy	83 (64.3)	7 (63.6)	76 (64.4)	
Chemotherapy	38 (29.5)	4 (36.4)	34 (28.8)	
Anti-CTLA-4	8 (6.2)	0	8 (6.8)	
PD-L1 testing^*^	71 (55.0)	7 (63.6)	64 (54.2)	0.75
PD-L1 result^¶^				
≧1	37 (52.1)	4 (57.1)	33 (51.6)	0.78
≧10	27 (38.0)	2 (28.6)	25 (39.1)	0.59
Visceral metastasis	70 (54.3)	4 (36.4)	66 (55.9)	0.34
Liver	25 (19.4)	2 (18.2)	23 (19.5)	0.99
Lung	46 (35.7)	1 (9.1)	45 (38.1)	0.10
Bone	25 (19.4)	2 (18.2)	23 (19.5)	0.99
Laboratory tests				
WBC ≧10,000/μl	102 (79.1)	6 (54.5)	96 (81.4)	0.70
Hgb <10 g/dl	49 (39.5)	9 (90.0)	40 (35.1)	0.001
NLR ≧5	49 (41.2)	5 (50.0)	44 (40.4)	0.74

CTLA-4, cytotoxic T-lymphocyte-associated protein 4; ECOG, Eastern Cooperative Oncology Group; ESRD, end-stage renal disease; Hgb, hemoglobin; ICI, immune checkpoint inhibitor; PD-1, programmed cell death protein 1; UCB, urothelial cancer of the bladder; UTUC, upper tract urothelial carcinoma; NLR, neutrophil to lymphocyte ratio; WBC, while blood cell count.

^*^PD-L1 immunohistochemistry testing used Dako 22C3 antibody.

^¶^Scoring by tumor proportion score (TPS) criteria.

**Table 2:** Patient profiles, treatment, response, and adverse events of ESRD group.

Patient	Age	Primary site	Therapy	Combination	Line	Response	OS (months)	Status	Hematologic AE	Other AE
1	58	Right renal pelvis	Atezolizumab	Paclitaxel	3	PD	8.05	AWD	Gr.4 neutropenia Gr.3 anemia Gr.1 thrombocytopenia	Gr.1 hepatitis Gr.1 anorexia Gr.1 fatigue
2	79	Left ureter	Pembrolizumab	–	1	PR	4.80	AWD	Gr.2 anemia	Gr.2 ascites
3	82	Left renal pelvis and ureter	Pembrolizumab	Gemcitabine	1	PD	0.72	DOD	Gr.3 neutropenia Gr.3 anemia Gr.3 thrombocytopenia	–
4	69	Left renal pelvis	Pembrolizumab	–	1	PD	5.85	DOD	Gr.2 anemia	Gr.1 hepatitis Gr.3 anorexia
5	68	Right renal pelvis	Nivolumab	Gemcitabine	1	PR	12.16	AWD	Gr.3 neutropenia Gr.4 anemia Gr.2 thrombocytopenia	Gr.1 hepatitis Gr.3 ascites
6	63	Left renal pelvis	Nivolumab	–	3	PD	0.23	DOD	Gr.2 anemia Gr.1 thrombocytopenia	Gr.4 CRS
7	45	Right renal pelvis and bladder	Atezolizumab	Paclitaxel	2	SD	19.68	AWD	Gr.4 anemia	–
8	65	Right renal pelvis	Pembrolizumab	–	2	PR	27.17	AWD	Gr.2 neutropenia Gr.2 anemia Gr.1 thrombocytopenia	Gr.2 eczema
9	66	Right renal pelvis	Atezolizumab	–	1	PR	15.54	AWD	Gr.4 neutropenia Gr.4 anemia Gr.1 thrombocytopenia	Gr.3 TB peritonitis Gr.4 TEN
10	74	Bladder	Pembrolizumab	–	2	PR	14.26	AWD	Gr.3 anemia Gr.1 thrombocytopenia Gr.2 anorexia	Gr.2 fatigue
11	35	Bladder	Pembrolizumab	–	1	PR	4.63	AWD	Gr.2 neutropenia Gr.2 anemia Gr.1 thrombocytopenia	–

OS, overall survival; AE, adverse event; PD, progressive disease; PR, partial response; SD, stable disease; AWD, alive with disease; DOD, dead of disease; Gr, grade; CRS, cytokine release syndrome; TB, tuberculosis; TEN, toxic epidermal necrolysis.

### Treatment-Related AEs

**Figure 1:**
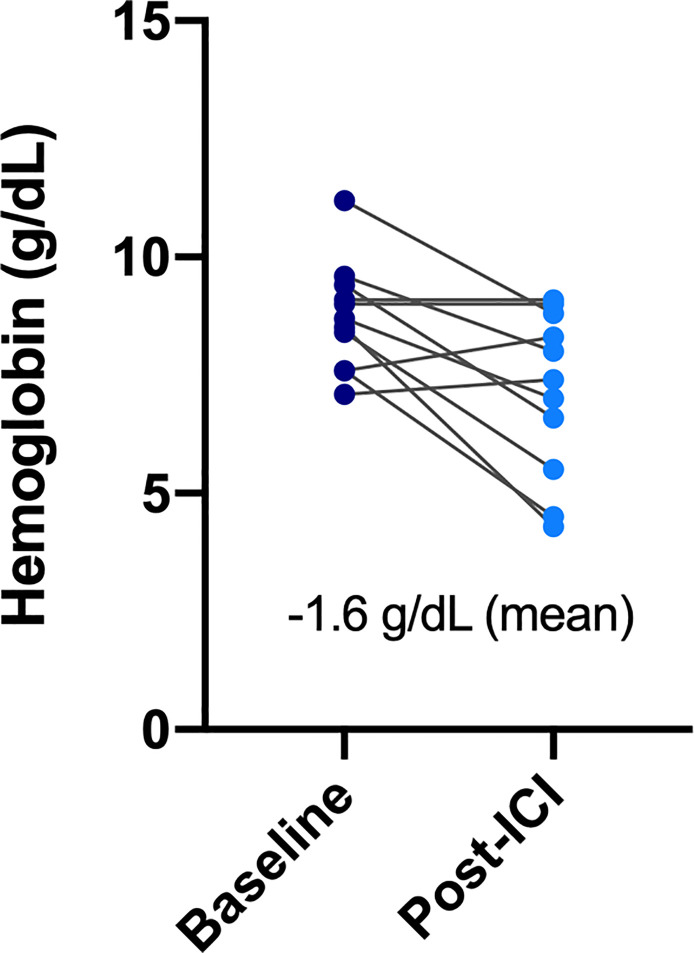
Change of hemoglobin before and after ICIs administration in the ESRD group.

All patients in the ESRD group experienced at least one treatment-related AE during the treatment period, and seven of them (63.6%) had high-grade (grade ≥3) AEs (**Table 3**). AEs of all grades included hematologic toxicity (neutropenia 54.5%; anemia 100%; and thrombocytopenia 72%), hepatitis (27.3%), fatigue (18.2%), anorexia (27.3%), and dermatologic toxicity (18.2%). Regarding hematologic toxicity, four patients (36.4%) had grade 3 neutropenia or higher, six (54.5%) had grade 3 anemia or higher, and one (9.1%) had grade 3 thrombocytopenia or higher. However, given the nature of defective function on hematopoiesis for patients with ESRD, the median baseline hemoglobin (Hb) of ESRD group was 8.75 g/dl. The low level of baseline Hb in ESRD group can actually be categorized in CTCAE grade 2 anemia, indicating that any decline of Hb will classified into grade 3 anemia. Although a considerable number of grade 3–4 anemia were observed in the ESRD group, the decrease in mean Hb between baseline and post-ICI administration was 1.6 g/dl, which was not substantially significant (**Figure 1**). For one who developed toxic epidermal necrolysis (TEN), a grade 4 dermatologic AE was recorded. Two patients presented with refractory ascites after receiving a PD-1 inhibitor. The ascites subsided after ICI usage was discontinued and recurred again after the re-administration of ICIs for disease relapse. One patient had disseminated tuberculosis reactivation. A cytokine release syndrome (CRS)-like syndrome was observed in one patient who presented with intermittent spiking fever and respiratory failure after receiving a PD-1 inhibitor.

**Table 3:** Adverse events in ESRD and non-ESRD group.

Adverse events	ESRD (%)	Non-ESRD (%)	*p* value
Any grade	11 (100)	84 (71.2)	0.04
Grade 3/4	7 (63.6)	42 (35.6)	0.07
Neutropenia	6 (54.5)	27 (22.9)	0.02
Grade 3/4	4 (36.4)	10 (8.5)	0.004
Anemia	11 (100)	54 (45.8)	0.001
Grade 3/4	6 (54.5)	32 (27.1)	0.07
Thrombocytopenia	8 (72.0)	43 (36.4)	0.02
Grade 3/4	1 (9.1)	16 (13.6)	0.68
Hepatitis	3 (27.3)	34 (28.8)	0.91
Fatigue	2 (18.2)		
Anorexia	3 (27.3)		
Skin^*^	2 (18.2)		
AE of specific interest			
Ascites	2 (18.2)		
TB reactivation	1 (9.1)		
TENS	1 (9.1)		
CRS-like syndrome	1 (9.1)		

AE, adverse event; TB, tuberculosis; TENS, toxic epidermal necrolysis; CRS, cytokine release syndrome; ESRD, end-stage renal disease.

^*^One TENS classified as grade 4 dermatologic toxicity.

We also compared the incidence of all grade AE and hematologic AE between ESRD and non-ESRD groups. As shown in **Table 3**, patients with ESRD on ICIs treatment had a higher incidence of all grade of neutropenia (54.5 vs. 22.9%, *p* = 0.02), anemia (100 vs. 45.8%, *p* = 0.001) and thrombocytopenia (72.0 vs. 36.4%, *p* = 0.02) than non-ESRD patients. Except for hematologic toxicity, there was no new additional safety concerns emerged from this comparative study between ESRD and non-ESRD group.

### Treatment Responses

The objective response rate (ORR) was significantly higher in the ESRD group than in the non-ESRD group (54.5 vs. 28.8%, p = 0.09). In terms of the disease control rate (DCR), the ESRD group benefited more (63.6%) than the non-ESRD group did (50.0%); in the ESRD group, six patients achieved partial response (54.5%), and one patient achieved stable disease status (9.1%). All details are provided in **Table 4**.

**Table 4:** Treatment response.

	ESRD (%)	Non-ESRD (%)	*p* value
Complete response (CR)	0	15 (12.7)	
Partial response (PR)	6 (54.5)	19 (16.1)	
Stable disease (SD)	1 (9.1)	25 (21.2)	
Progressive disease (PD)	4 (36.4)	59 (50.0)	
Overall response rate (ORR)	6 (54.5)	34 (28.8)	0.09
Disease control rate (DCR)	7 (63.6)	59 (50.0)	0.53

ESRD, end-stage renal disease.

### Survival Outcomes

**Figure 2:**
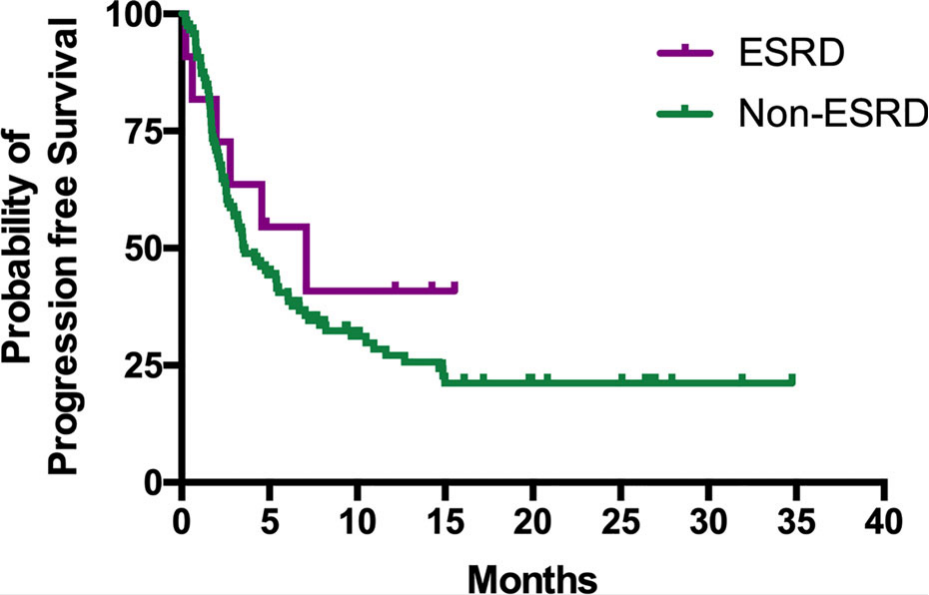
Kaplan–Meier curves of PFS for mUC patients with or without ESRD receiving ICIs.

**Figure 3:**
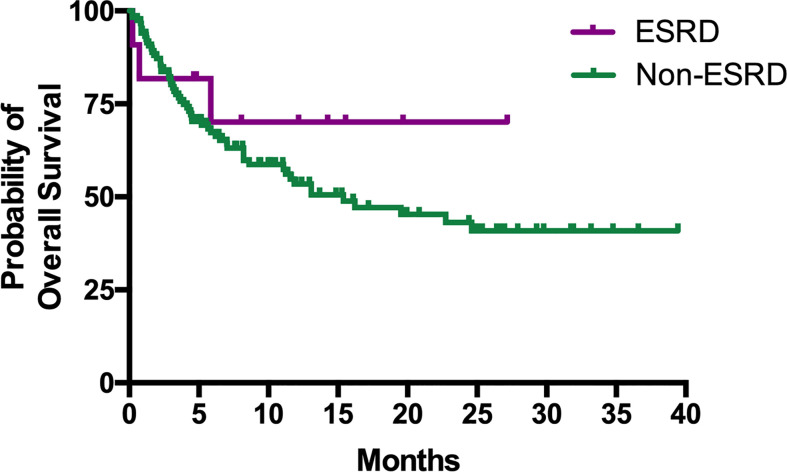
Kaplan–Meier curves of OS for mUC patients with or without ESRD receiving ICIs.

The median PFS of patients in the ESRD and non-ESRD groups was 7.1 and 3.5 months, respectively (p = 0.42; the PFS curve is plotted in **Figure 2**). The median OS of patients in the ESRD group was not reached and was 15.4 months in the non-ESRD group (the OS curve is plotted in **Figure 3**). In the univariate analysis of OS, the prognostic factors included ECOG (≥2 vs. <1; HR: 1.96; 95% CI: 1.06–3.65; p <0.03), leukocytosis (≥10,000/μl vs. <10,000/μL; HR: 3.80; 95% CI: 2.22–6.51; p <0.001), anemia (<10 g/dl vs. ≥10 g/dl; HR: 2.41; 95% CI: 1.43–4.04; p = 0.001) and NLR (≥5 vs. <5; HR: 3.93; 95% CI: 2.29–6.77; p <0.001). In the univariate analysis, a trend of survival benefits was observed for patients without liver metastasis (HR: 1.65; 95% CI: 0.92–2.98; p = 0.09) and without lung metastasis (HR: 1.59; 95% CI: 0.95–2.66; p = 0.08). After adjustments were made for all potential prognostic factors in the multivariate analysis, the only independent factor was leukocytosis (HR: 2.63; 95% CI: 1.23–5.63; p = 0.01) and NLR (HR: 2.91; 95% CI: 1.30–6.53; p = 0.01). All details are presented in **Table 5**.

**Table 5:** Univariate and multivariate analysis of overall survival.

Characteristics	Median OS	Univariate		Multivariate	
	(month)	HR (95% CI)	*p* value	HR (95% CI)	*p* value
Age (year)			0.50		0.19
<65	13.1	1		1	
≧65	22.7	0.84 (0.50–1.40)		0.65 (0.35–1.23)	
Gender			0.59		0.52
Female	15.4	1		1	
Male	19.5	0.87 (0.52–1.45)		1.26 (0.62–2.55)	
Primary tumor			0.21		0.28
UCB	22.7	1		1	
UTUC	11.9	1.42 (0.82–2.47)		1.45 (0.74–2.85)	
ECOG			0.03		0.35
0–1	16.2	1		1	
≧2	4.4	1.96 (1.06–3.65)		1.47 (0.66–3.29)	
ICI sequence					
1st line	19.5	1	0.37	1	0.14
2nd line	16.2	0.95 (0.46–1.95)	0.89	2.22 (0.93–5.28)	0.07
3rd line or later	5.2	1.69 (0.79–3.61)	0.18	0.83 (0.28–2.43)	0.73
ICI type			0.68		0.78
Anti-PD-1	15.4	1		1	
Anti-PD-L1	19.5	0.89 (0.52–1.54)		0.91 (0.45–1.83)	
Treatment partner					
Monotherapy	13.1	1	0.83	1	0.64
Chemotherapy	NR	0.84 (0.47–1.52)	0.57	0.85 (0.43–1.69)	0.64
Anti-CTLA-4	13.1	1.05 (0.41–2.68)	0.92	0.57 (0.15–2.12)	0.40
Visceral metastasis			0.29		0.91
No	16.2	1		1	
Yes	13.4	1.33 (0.81–2.29)		0.94 (0.29–3.05)	
Liver metastasis			0.09		0.30
No	22.7	1		1	
Yes	8.2	1.65 (0.92–2.98)		1.53 (0.68–3.42)	
Lung metastasis			0.08		0.17
No	NR	1		1	
Yes	8.6	1.59 (0.95–2.66)		1.95 (0.75–5.07)	
Bone metastasis			0.49		0.42
No	15.4	1		1	
Yes	8.6	1.24 (0.67–2.31)		0.72 (0.33–1.60)	
Leukocytosis			<0.001		0.01
WBC <10,000/μl	24.6	1		1	
WBC ≧10,000/μl	3.9	3.80 (2.22–6.51)		2.63 (1.23–5.63)	
Anemia			0.001		0.38
Hgb ≧10 g/dl	22.7	1		1	
Hgb <10 g/dl	4.4	2.41 (1.43–4.04)		1.45 (0.64–3.28)	
Neutrophil to lymphocyte ratio			<0.001		0.01
NLR <5	NR	1		1	
NLR ≧5	4.1	3.93 (2.29–6.77)		2.91(1.30–6.53)	

CTLA-4, cytotoxic T-lymphocyte-associated protein 4; ECOG, Eastern Cooperative Oncology Group; ESRD, end-stage renal disease; Hgb, hemoglobin; ICI, immune checkpoint inhibitor; OS, overall survival; PD-1, programmed cell death protein 1; NLR, neutrophil to lymphocyte ratio; NR, non-reach; UCB, urothelial cancer of the bladder; UTUC, upper tract urothelial carcinoma; WBC, while blood cell count.

## Discussion

The present study reports the treatment experience of 11 consecutive patients with ESRD who received ICIs for mUC. Although some unexpected AEs occurred, generally, in patients with ESRD, the ICIs were well tolerated without additional toxicity. Furthermore, the major efficacy endpoints of ORR, PFS, and OS suggested benefits of ICI use in patients with ESRD. To our knowledge, this is the largest case series on the safety and efficacy of ICIs for patients with cancer who require maintenance hemodialysis. Our real-world data indicate that the administration of ICIs may be beneficial in such difficult treatment scenarios.

A few case reports and case series had examined the efficacy and safety of administrating ICIs in patients with ESRD on dialysis. In reviewing literature, only 41 patients had been reported; most of them were metastatic melanoma, NSCLC and renal cell carcinoma (RCC), only five cases were mUC (13–31) (**Table 6**) Vitale et al. reported eight ESRD patients with metastatic RCC who received dialysis (seven on hemodialysis, one on peritoneal dialysis) and nivolumab as cancer treatment. Only two patients (25%) experienced grade 3 AEs (diarrhea, asthenia, and anorexia), and five patients (62.5%) had grade 1–2 AEs, including cutaneous toxicities, anorexia, diarrhea, nausea, vomiting, arthralgia, and hematologic toxicities. These irAEs were appropriately managed with systemic corticosteroid and symptomatic treatment (15). Strohbehn et al. presented a brief report of treatment response and side effects in 19 ESRD patients received ICI therapy. However, the study population were quite heterogeneous in cancer types (six genitourinary cancer, three melanoma, three merkel cell carcinoma, three head and neck cancer), ICI regimen (90% anti-PD-1/PD-L1, 5% anti-CTLA-4 and 5% combined anti-PD-1/CTLA-4), and dialysis modality (79% hemodialysis, 21% peritoneal dialysis), which limited to achieve a definite conclusion (32). Compared with previous reports, our study revealed more hematologic AEs, 36.4% of which were grade 3–4 neutropenia. However, a standard chemotherapy regimen, either of gemcitabine plus cisplatin or MVAC (methotrexate, vinblastine, doxorubicin, and cisplatin), caused more than 70% of patients to experience grade 3–4 neutropenia (3). Given concerns related to neutropenia and risk of infection, ICI is a safe treatment for patients with mUC and ESRD.

**Table 6:** Summary of 41 published cases of the use of immune checkpoint inhibitors in dialysis patients.

Reference	n	Age	Dialysis	Cancer	ICI	Response	Toxicity
Cavalcante et al. (14)	2	56,69	HD	Melanoma	Ipilimumab	CR (1),PR (1)	G2 fatigue, G1-2 pruritus, G3 pemphigoid rash
Boils et al. (16)	1	74	HD^*^	NSCLC-SCC	Nivolumab	NA	Renal allograft rejection (3 doses)
Ong et al. (17)	1	76	HD^*^	Melanoma	Nivolumab	PR	Renal allograft rejection (8 days)
Carlo et al. (18)	1	77	HD	mRCC	Nivolumab	PR	Pseudo-progression with respiratory failure
Chang et al. (19)	1	63	HD	Melanoma	Pembrolizumab	CR	G1 fatigue
Lipson et al. (20)	1	57	HD^*^	Cutaneous SCC	Pembrolizumab	PR (85% reduction)	Renal allograft rejection (2 months)
Spain et al. (21)	1	48	HD^*^	Melanoma	Ipilimumab (1) Nivolumab (2)	PR	Renal allograft rejection (8 days of nivolumab)
Alhamad et al. (22)	1	68	HD^*^	Melanoma	Ipilimumab (1) Pembrolizumab (2)	Progression (1) NA (Pembrolizumab)	Renal allograft rejection (3 weeks of pembrolizumab)
Jose et al. (23)	1	40	HD/PD^*^	Melanoma^†^	Ipilimumab	Progression	Renal allograft rejection (after two cycles)
Tabei et al. (24)	1	49	HD	RCC	Nivolumab	PR	No AEs
Boyle et al. (25)	1	57	HD	Melanoma^‡^	Nivolumab	PR	No AEs
Park and Daniels (26)	4	66–71	HD (3) PD (1)	RCC (2) Cutaneous SCC (2)	Nivolumab (2) Pembrolizumab (2)	SD (1), PR (3)	G2 rash, G2 fatigue G3 pneumonitis, G4 encephalitis^¶^
Ishizuka et al. (27)	1	66	HD	NSCLC-SCC	Pembrolizumab	PR	G1 rash
Ansari et al. (28)	1	72	HD	RCC	Nivolumab	PR	No G2-4 AEs
Cheun et al. (13)	3	64–68	HD	RCC (2) Renal pelvic UC (1)	Nivolumab (2) Atezolizumab (1)	PR (1), SD (1), Progression (1)	G2 pneumonitis
Vitale et al. (15)	8	51–77	HD (7) PD (1)	RCC (8)	Nivolumab	PR (1), SD (5), Progression (2)	G2 Nausea, G1 Vomiting, G2-3 Diarrhea G2-3 Anorexia, G1-3 Asthenia, G1 Arthralgia G1-2 Cutaneous, G1-2 Hematologic
Parisi et al. (29)	1	NA	HD	UC^§^	Atezolizumab	PR	G1 itching, G1 asthenia G1 nausea, G1 dysgeusia, G1 constipation
Osmán-García et al. (30)	3	60–77	HD (2) PD (1)	RCC	Nivolumab	PR (2), PD (1)	No G2-4 AEs
Hirsch et al. (31)	8	35–83	HD (7) PD (1)	UC (3), HCC (1), CCA (1), HL (1), NET (1), RCC (1)	Pembrolizumab (4) Nivolumab (3)^||^ Iipilimumab (1)^||^ Atezolizumab (1)	SD (3) Progression (5)	Dermatitis (1) Renal allograft rejection (1)
Current study	11	35–82	HD	UC (11)	Pembrolizumab (6) Nivolumab (2) Atezolizumab (3)	PR (6), SD (1) Progression (4)	G1-4 cytopenia, G1 hepatitis, G2-3 ascites G4 CRS, G3 TB peritonitis, G4 TEN G1-3 anorexia, G1-2 fatigue, G2 eczema

n, case number; NA, not available; HD, hemodialysis; PD, peritoneal dialysis; NSCLC, non-small cell lung cancer; SCC, squamous cell carcinoma; RCC, renal cell carcinoma; UC, urothelial carcinoma; HCC, hepatocellular carcinoma; CCA, cholangiocarcinoma; HL, Hodgkin lymphoma; NET, neuroendocrine tumor; ICI, immune checkpoint inhibitor; CR, complete response; PR, partial response; SD; stable disease; G, grade; AE, adverse event; CRS, cytokine release syndrome; TB, tuberculosis; TEN, toxic epidermal necrolysis.

^*^Dialysis dependence after renal graft rejection.

^†^Choroid melanoma.

^‡^Donor derived melanoma.

^§^Bladder sarcomatoid carcinoma.

^||^One patient received both nivolumab and ipilimumab.

^¶^In this case report, one patient died from possible treatment-related causes.

We also reported some notable irAEs in this study. A 65-year-old woman had disseminated tuberculosis reactivation and TEN after anti-PD-L1 administration. The patient fully recovered from TEN after systemic steroid administration and intensive skin care, and her tuberculosis was appropriately controlled by anti-tuberculosis agents. It is worthwhile to highlight the relationship between ICI use and TB reactivation. Barber et al. hypothesized that ICIs may boost T_H_1 function and increase the level of interferon γ-producing *Mycobacterium tuberculosis*-specific CD4 T-cells in the blood (33). The pathogenesis of TEN is also related to cell-mediated cytotoxic reactions and the clonal expansion of drug-specific T-cells with cytotoxicity against keratinocytes directly and indirectly through the recruitment of other cells (34). Cavalcante et al. reported that a patient with ESRD developed a grade 3 pemphigoid rash and bullous lesion after ipilimumab administration, achieving a complete response (14). Further studies are required to clarify the incidence of severe dermatologic irAEs in patients with ESRD and to elucidate the relationship between the intensity of cell-mediated cytotoxic reactions and the durable response rate.

One patient in our study presented with daily spiking fever, hypotension, altered mental status, hypoxia, and respiratory failure after administration of the first cycle of anti-PD-1 treatment. The clinical manifestation was thought to be severe sepsis but also resembled an unusual form of CRS, an inflammatory systemic disorder resulting from an overwhelming elevation of cytokine levels and T-cell engagement and proliferation. CRS severity can range from mild symptoms to a fulminant disease with multiple organ failure and death. CRS has been observed to be triggered by several monoclonal antibodies, systemic interleukin-2, and more recently, the CD19-CD3 chimeric antigen receptor T-cell therapy (35). A few case reports have detailed life-threatening CRS in patients after the administration of ICIs, with occurrences ranging from cycles 1 to 17 (36–39). The culprit medications were anti-PD-1 and anti-LAG-3. Alexander et al. reported the case of a patient with stage IV melanoma who received nivolumab on cycle 17 and had a CRS episode; it was controlled by tocilizumab initially, but the patient died 6 weeks later because of another CRS episode (39). Seth et al. also reported a patient with alveolar soft part sarcoma who received nivolumab and had a CRS event that was resolved by tocilizumab and corticosteroids (38). Although CRS is an uncommon complication associated with ICIs, early recognition and prompt management of CRS is crucial owing to its high mortality risk.

Among patients with ESRD in this report, ICIs conferred a significantly higher ORR and better DCR on patients with ERSD than those without. The response rate benefits reflect the trends of better PFS and median OS. Our results showed that the efficacy of ICIs for patients with ESRD was not inferior to that for patients without ESRD. A possible explanation of the superior antitumor efficacy of ICIs may be related to pharmacokinetics. Renal failure or hemodialysis seems to have no effect on the pharmacokinetics of ICIs, possibly because the clearance of ICIs is governed by numerous physiological mechanisms; this clearance predominantly occurs through nonspecific degradation within plasma and tissues. This nonspecific route of degradation reduces the influence of age, hepatic impairment, and renal failure on clearance (40). Considering the large molecular weights of ICIs (nivolumab: 146 kDa; ipilimumab: 148 kDa; pembrolizumab: 149 kDa; atezolizumab: 145 kDa), which cannot penetrate dialysis pores, drug removal and elimination through hemodialysis are unlikely (13). The pharmacokinetic characteristics of ICIs, which are unaffected by renal failure and hemodialysis, were also demonstrated by a similar incidence of AEs among patients in the ESRD and non-ESRD groups.

This study had some inevitable limitations owing to its retrospective nature; furthermore, it was limited by the relatively small sample size of the ESRD group. However, it is difficult to conduct a prospective clinical trial through recruiting patients with advanced UC or mUC to receive ICIs. The difficulty is not simply due to sample size; additionally, ESRD may develop during the treatment period among such patients with UC. Finally, the study had unpreventable bias in terms of the choice of ICIs being governed by physicians’ decisions, patients’ financial considerations, and the instructions of the National Health Insurance system in Taiwan. However, our results demonstrated that the administration of ICIs in patients with ESRD resulted in them having a better survival trend than did patients without ESRD, and no notable safety concerns arose.

In conclusion, our study revealed that administration of ICIs in patients with mUC and ESRD demonstrated a modest antitumor activity, and should be used with caution for increasing risk of hematologic toxicity. Further confirmatory studies are required to validate our findings.

## Data Availability Statement

The raw data supporting the conclusions of this article will be made available by the authors, without undue reservation.

## Ethics Statement

The studies involving human participants were reviewed and approved by Institutional Review Board of Chang Gung Medical Foundation. Written informed consent for participation was not required for this study in accordance with the national legislation and the institutional requirements.

## Author Contributions

M-CK analyzed and interpreted data, prepared the tables, and wrote the original manuscript. Y-LS designed the conceptualization and methodology, prepared the figures, and reviewed and edited the manuscript. P-JS, C-CH, H-LL, T-JC, S-HL, C-CW, T-TL, Y-TC, and C-HK contributed the resources. All authors contributed to the article and approved the submitted version.

## Conflict of Interest

The authors declare that the research was conducted in the absence of any commercial or financial relationships that could be construed as a potential conflict of interest.
