# Supplementary material for: Safety and Efficacy of Immune Checkpoint Inhibitors for Patients With Metastatic Urothelial Carcinoma and End-Stage Renal Disease: Experiences From Real-World Practice
Source: Front Oncol. 2020 Nov 27;10:584834. doi: 10.3389/fonc.2020.584834 (PMC7729012; doi:10.3389/fonc.2020.584834)
Supplement: Supplementary file 1 [file DataSheet_1.docx]

Supplementary Material

# Supplementary Table 1: Definition of CTCAE hematologic adverse events

|  | Neutrophil count decreased | Anemia (Hemoglobin) | Platelet count decreased |
| --- | --- | --- | --- |
| Grade 1 | 1500 /mm^3^ - LLN  1.5 * 10^-9^ /L - LLN | 10.0 g/dL - LLN  6.2 mmol/L - LLN | 75000 /mm^3^ - LLN  75.0 * 10^-9^ /L - LLN |
| Grade 2 | 1000 - 1500 /mm^3^  1.0 - 1.5 * 10^-9^ /L | 8.0 - 10 g/dL  4.9 - 6.2 mmol/L | 50000 - 75000 /mm^3^  50.0 - 75.0 * 10^-9^ /L |
| Grade 3 | 500 - 1000 /mm^3^  0.5 - 1.0 * 10^-9^ /L | < 8.0 g/dL  < 4.9 mmol/L  Transfusion indicated | 25000 - 50000 /mm^3^  25.0 - 50.0 * 10^-9^ /L |
| Grade 4 | < 500 /mm^3^  < 0.5 * 10^-9^ /L | Life-threatening consequences  Urgent intervention indicated | < 25000 /mm^3^  < 25.0 * 10^-9^ /L |
| Grade 5 | - | Death | - |

Abbreviation: LLN, lower limit of normal
